# Self-administered subcutaneous medroxyprogesterone acetate for improving contraceptive outcomes: a systematic review and meta-analysis

**DOI:** 10.1186/s12905-021-01495-y

**Published:** 2021-10-09

**Authors:** Ashraf Nabhan, Farida Elshafeey, Luna Marion Mehrain, Rita Kabra, Amal Elshabrawy

**Affiliations:** 1 Department of Obstetrics and Gynecology, Faculty of Medicine, Ain Shams University, Abbassia, Cairo, Egypt; 2 Egyptian Center for Evidence Based Medicine, Cairo, Egypt; 3 International Planned Parenthood Federation, London, UK; 4 World Health Organization, Geneva, Switzerland

**Keywords:** Depo medroxyprogesterone acetate, Self-administration, Family planning, Long-Acting Reversible Contraception

## Abstract

**Background:**

Subcutaneous depot medroxyprogesterone acetate is an easy-to-use injectable contraceptive. A trained person can administer it, including women through self-injection. The objective of this systematic review and meta-analysis was to assess the effectiveness and safety of self-injection versus provider-administered subcutaneous depot medroxyprogesterone acetate for improving continuation of contraceptive use.

**Methods:**

We searched for randomized controlled trials on November 1, 2020 in Cochrane Central Register of Controlled Trials, MEDLINE, CINAHL, Embase, Web of Science, Scopus, Open Grey, clinical trials registries, and reference lists of relevant studies. We did not impose any search restrictions. We included randomized trials comparing self- versus provider-administered subcutaneous depot medroxyprogesterone acetate. Two authors independently screened trials, extracted data, and assessed the risk of bias in the included studies. We used risk ratio and 95% confidence intervals for dichotomous outcomes.

**Results:**

We identified 3 randomized trials (9 reports; 1264 participants). The risk of bias in the included studies was low except for performance bias and detection bias of participant-reported outcomes in unmasked trials. Self-administration, compared to provider-administration, increased continuation of contraceptive use (risk ratio 1.35; 95% confidence intervals 1.10–1.66); moderate-certainty evidence). Self-injection appears to be making more of an impact on continuation for younger women compared to women 25 years and older and on women living in low and middle income compared to high income countries. There was no subgroup difference by the type of care provider (community health worker vs. clinic-based provider).

**Conclusions:**

Self-injection of subcutaneous depot medroxyprogesterone acetate probably improves continuation of contraceptive use. The effects on other outcomes remain uncertain because of the very low certainty of evidence.

**Supplementary Information:**

The online version contains supplementary material available at 10.1186/s12905-021-01495-y.

## Background

Depo-Medroxyprogesterone Acetate (DMPA), a synthetic progestin derived from 17-hydroxyprogesterone, acts as a long-acting reversible contraceptive. Most women who want a safe, effective, and reversible method can use DMPA injectable contraception [1].

While users, traditionally, receive DMPA by intramuscular injection every 13 weeks in a dose of 150 mg, it turns out that subcutaneous administration of a lower dose of DMPA (DMPA-SC) is an effective alternative [2]. Studies demonstrated that a single injection of DMPA-SC (104 mg medroxyprogesterone acetate/0.65 mL) provided immediate suppression of ovulation and consistently suppressed ovulation over 13 weeks, with the earliest return to ovulation at 15 weeks. This consistent suppression of ovulation with this 30% lower dose, was independent of body mass index or race. The subcutaneous route, compared to intramuscular route, provided lower peak levels, lower overall dose and, apparently, more stable sustained blood levels [2]. The subcutaneous route has been shown to have comparable efficacy and safety to the intramuscular route [3].

From a practical and a programmatic perspective, the subcutaneous route provides a unique opportunity of self-administration [4]. DMPA-SC is available as a pre-filled glass syringe or as a pre-filled, single-use, non-reusable delivery system. The user-friendly design of DMPA-SC means that any trained person can administer it, including health workers, pharmacists, and even women themselves through self-injection [5–7].

Self-injection empowers women to meet their family planning needs. Encouraging self-administration of injectable contraception can help women build their health assets, make health care products and practices more accessible, and reinforce the internationally agreed upon human right to good health and self-determination. Self-injection has the potential of reducing discontinuation of contraceptive use, thus avoiding unwarranted pregnancy. Self-injection probably saves time and expenses, a factor that is particularly relevant in low resource settings, in humanitarian crisis where health infrastructure is disrupted, or as part of preparedness [4]. The current coronavirus pandemic and lockdown in many countries is a real-life example.

On the other hand, some women might prefer having providers administer injections due to factors like fear of needles or provider expertise [8].

Reducing discontinuation of contraceptive methods, thus avoiding unintended pregnancy, remains a challenge in many countries. There is little rigorous updated synthesized evidence to enable women and care providers to make well informed decisions regarding self-administered DMPA-SC.

We conducted this systematic review and meta-analysis to assess the benefits and harms of self-administered DMPA-SC for improving continuation of contraceptive use, women’s satisfaction and avoiding unintended pregnancy.

## Methods

### Protocol and registration

We prepared the protocol following the methodological standards of Cochrane handbook [9]. We prospectively registered the protocol on PROSPERO (international prospective register of systematic reviews) (registration number CRD42018097388). The full text of the protocol is available in an open access registry [10].

We reported the full review using the Preferred Reporting Items for Systematic reviews and Meta-Analyses (PRISMA) standards [11].

### Eligibility criteria

We included published randomized controlled trials that recruited women attending family planning clinic to initiate, restart or continue DMPA and compared self- administered at home with clinic-administered DMPA-SC by a healthcare provider. Our primary outcome was contraceptive continuation at 12 months. Secondary outcomes included contraceptive failure (pregnancy), satisfaction, serious adverse events, and other adverse events at 12 months of follow-up.

### Information sources

A comprehensive literature search was initially conducted on March 1, 2019, and then on November 1, 2020. We imposed no language or other restrictions on any of the searches. We searched bibliographic databases (Cochrane Central Register of Controlled Trials (CENTRAL), MEDLINE, CINAHL, and Embase), citation indexes (Web of Science and Scopus), and one grey-literature database (opengrey.eu). We searched clinical trial registries (ClinicalTrials.gov and the World Health Organization International Clinical Trials Registry Platform) to identify ongoing trials. We hand searched reference lists and explored the cited-by logs of identified studies and previously published reviews.

### Search

The search strategy was designed by a search expert with input from the authors. We used the following search strategy for CENTRAL (“Medroxyprogesterone Acetate” or “Depo-Medroxyprogesterone Acetate” or “Depo Medroxyprogesterone Acetate” or “Depo-Provera” or “Depo Provera” or “Provera” or “Sayana” or “Depo subQ Provera”: title,abstract,keyword OR MeSH descriptor: [Medroxyprogesterone]) AND (MeSH descriptor: [Self Administration] or “self”: title,abstract,keyword). The detailed exact strategy for each database searched is provided in Additional file 1.

### Study selection

Two authors (FS and AE) independently screened all titles and abstracts for eligibility. We retrieved and assessed the full text of all the studies that potentially met our eligibility criteria during screening. Both authors independently assessed each full-text article based on the eligibility criteria described above. Disagreements regarding trial eligibility were resolved by consensus and finally resolved by a third author (AN).

### Data collection process

For eligible studies, two authors (FS and AE) extracted the data in duplicates using an offline electronic form. We resolved discrepancies through discussion. We entered the data into Review Manager Software [12] and checked them for accuracy. We contacted authors of the original reports to provide further details regarding unclear or missing data.

### Data items

We extracted study design, description of included participants, description of the intervention and comparators, outcomes, trial registration, and funding sources.

### Risk of bias in individual studies

We assessed the risk of bias using the criteria recently outlined in the Cochrane Handbook for Systematic Reviews of Interventions [9]. Seven domains related to risk of bias were assessed in each included trial: random sequence generation; allocation concealment; blinding of participants and personnel; blinding of outcome assessment; incomplete outcome data; selective reporting; and other bias. Review authors’ judgments were categorized as “low,” “high,” or “unclear” risk of bias. Two authors independently assessed the risk of bias in each trial. We resolved any differences of opinion regarding assessment of risk of bias by discussion.

### Summary measures

An intent-to-treat analysis, including all randomized women, was performed. All studies were parallel group assignment. We did not include any multiple arms, cluster, or crossover trials. For dichotomous data, we presented results as summary risk ratio (RR) with 95% confidence intervals (CI).

### Synthesis of results

Fixed-effect meta-analysis was performed to combine data of trials that were judged to be sufficiently similar in terms of intervention, populations, and methods. Substantial statistical heterogeneity, defined as I^2^ statistic ≥ 50% or P < 0.1, was investigated and a random-effects meta-analysis was performed only if an average treatment effect across trials was considered clinically meaningful. The number needed to treat (NNT) for benefit or harm with the 95% CI was calculated for outcomes for which there was a statistically significant difference [9].

### Risk of bias across studies

The assessment of publication bias was not possible because we only included three studies in the meta-analyses that pooled data [9].

### Additional analyses

Substantial heterogeneity, as defined in the protocol, was thoroughly investigated based on the prespecified methods. We performed the planned subgroup analysis by country income: High income countries (HIC) versus low-middle income countries (LMIC) (as defined by the World bank) and by type of care provider (community health worker versus clinic-based provider). We assessed subgroup differences by interaction tests available within Review Manager. Results of the subgroup analyses were reported by mentioning the Chi^2^ statistic and P value, and the interaction test I^2^ value.

Sensitivity analyses were performed to explore robustness of pooled estimate using random effect model and fixed effect model for the outcome of continuation. Also, we performed sensitivity analyses to explore the effects of incomplete outcome data by conducting an available case versus a worst-case scenario analysis to evaluate robustness of results.

Statistical analysis was performed using Review Manager software version 5.3 [12].

We used the Grading of Recommendations, Assessment, Development and Evaluation (GRADE) approach to create the Summary of findings table. Briefly, GRADE uses study limitations, consistency of effect, imprecision, indirectness, and publication bias to assess the quality of the body of evidence for each outcome [13]. A summary of the intervention effect and a measure of quality for outcomes was produced using the GRADEpro GDT software [14]. One author (A.N.) conducted GRADE assessments and the decisions on downgrading. This was revised and approved by all other authors.

## Results

### Study selection

**Fig. 1:**
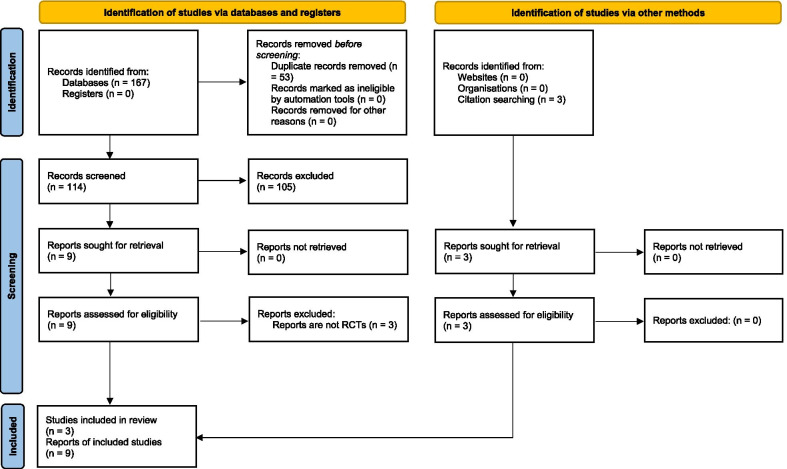
PRISMA study flow diagram

Our search identified 167 reports through database searches along with 3 additional reports identified through other resources. A total of 117 reports remained after removal of duplicates. We discarded 105 reports at the initial screening of the titles and abstracts because these clearly did not meet the eligibility criteria. We retrieved and assessed the full text of 12 reports. We excluded 3 reports of non-randomized studies. We included 9 reports of 3 studies [15–17]. Figure 1 shows the study selection process. We did not identify any ongoing trial.

### Study characteristics

#### Included studies

We summarized the characteristics of the included studies (country, participants, interventions, outcomes, study design, sample size, follow-up period) in Table 1.

**Table 1:** Characteristics of included studies

Study ID (Trial registration)	Study design	Number randomized	Age of included women	Trial arms	Duration of treatment (months)	Outcomes reported	Country
Burke 2018 NCT02293694	Open-label, parallel group, randomized clinical trial	731	18–40	Participants were randomized in a 1:1 ratio to self- or clinician administered subcutaneously DMPA 104 mg	12	Continuation, satisfaction, failure (unintended pregnancy), side effects	Malawi
Kohn 2018 NCT02509767	Multicenter, open label, randomized parallel group clinical trial	401	15–44	Participants were randomized in a 1:1 ratio to self- or clinician administered subcutaneously DMPA 104 mg	12	Continuation, satisfaction, failure (unintended pregnancy), side effects	USA
Beasley 2014 NCT01019369	Open label, parallel group, randomized clinical trial	132	18 or greater	Participants were randomized in a 2:1 ratio to self- or clinician administered subcutaneously DMPA 104 mg	12	Continuation, satisfaction, side effects	USA

### Methods and setting

We included three RCTs. The three trials included one multi-center trial in USA [15] and two single-site studies in USA [17] and Malawi [16].

### Participants

Studies included a total of 1264 participants who were randomized to self-administration (651 women) versus a provider administration (613 women). All studies included women, in their reproductive age, receiving DMPA-SC for contraception.

### Interventions

The three included studies randomized participants to receive self-administered or provider-administered DMPA-SC. The duration of follow-up was 12 months.

### Outcomes

All included trials reported continuation of injectable contraception at 12 months as patient reported [15–17]. Only one study verified DMPA use by measuring trough MPA levels in blood [15]. Two studies reported failure (unintended pregnancy) and satisfaction [16, 17], two studies reported other (non-serious) adverse events [15, 17], and three studies reported serious adverse events, if any [15–17].

### Risk of bias within studies

**Fig. 2:**
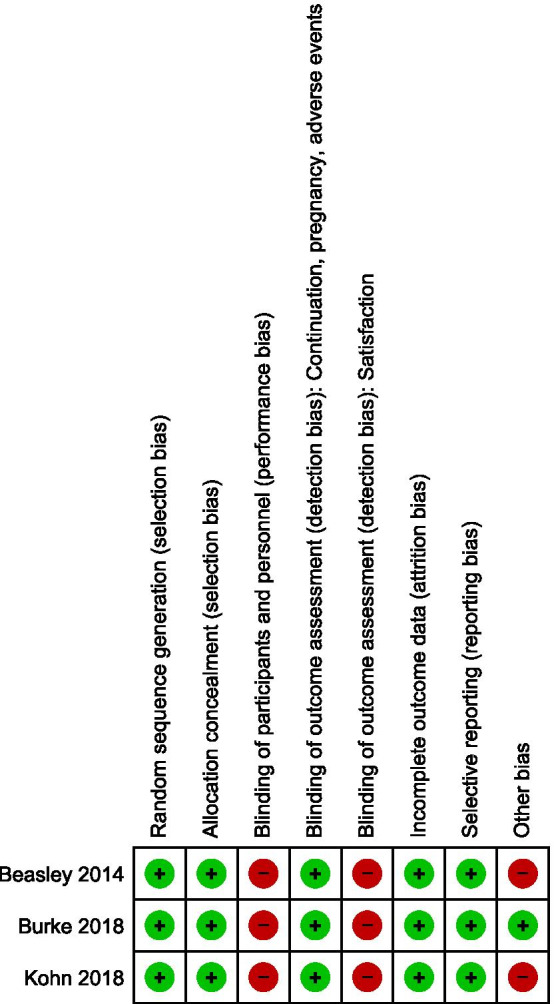
Risk of bias summary: review authors' judgements about each risk of bias item for each included study. Low risk:  , high risk:

We presented data on risk of bias of each study in Fig. 2. We made an outcome level assessment for detection bias since the lack of blinding may introduce bias in the measurement of women reporting of satisfaction. We judged the studies to be at high risk of performance bias due to the lack of blinding. The lack of blinding coupled with fixed block size is a potential source of selection bias in two of the included studies [15, 17]. Details and justifications for our judgements are provided in Additional file 1.

### Results of individual studies and Synthesis of results

Continuation of contraceptive use for 12 months was reported by the included RCTs (3 studies, 1261 women).

**Fig. 3:**
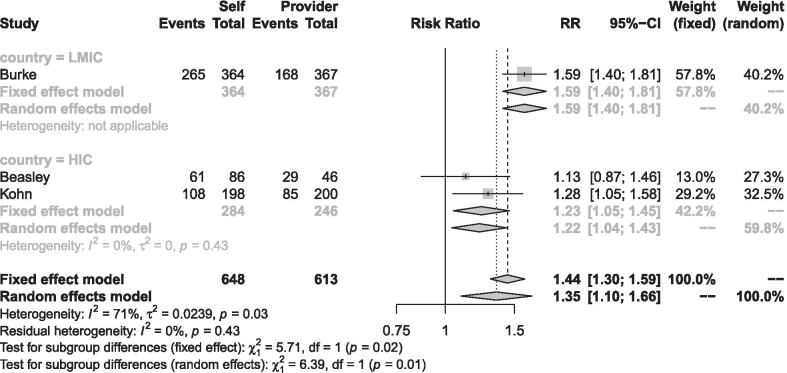
Self-administration versus provider-administration for continuation of contraceptive use at 12 months. *LMIC* low middle income countries, *HIC* high income countries

Self-administration, compared to provider-administration, improved continuation of contraceptive use (RR 1.3495 [1.0953; 1.6626]; P = 0.0049); moderate-certainty evidence), although there was substantial heterogeneity (Tau^2^ = 0.0239; I^2^ = 71.4% [3.1%; 91.6%]; P = 0.0301). We performed a sensitivity analysis using the fixed effect model that returned a Risk Ratio (M–H, Fixed, 95% CI) of 1.4401 [1.3023; 1.5924], P < 0.0001 (Fig. 3).

Further, we investigated the source of heterogeneity using the pre-specified subgroup analysis.

For the subgroup analysis HIC versus LMIC, the test for subgroup differences was significant (Chi^2^ = 5.71, df = 1, p = 0.0168), Fig. 3. In HIC (2 studies, 530 women), the Risk Ratio (M–H, random, 95% CI) was 1.22 [1.04, 1.43] (very low certainty evidence). The NNT-b is 10 (95% CI 5–53). In LMIC (1 study, 731 women), the Risk Ratio (M–H, random, 95% CI) was 1.59 [1.40, 1.81] (low certainty evidence), The NNT-b is 4 (95% CI 3–5).

For the subgroup analysis by the type of care provider (community health worker vs. clinic-based provider), the test for subgroup differences was not significant (Chi^2^ = 0.05, df = 1 (P = 0.83), I^2^ = 0%).

We also carried out a sensitivity analysis to assess the impact of attrition on the outcome of continuation, showing no change in the estimate of the effect.

Satisfaction at 12 months was reported by two studies.

In HIC (1 study, 398 women), Risk Ratio (M–H, Fixed, 95% CI) was 0.95 [0.84, 1.07] (very low certainty evidence). In LMIC (1 study, 731 women), Risk Ratio (M–H, Fixed, 95% CI) was 1.83 [1.61–2.07] (low certainty evidence). We did not pool the studies because there was substantial heterogeneity (Tau^2^ = 0.22; Chi^2^ = 55.34, df = 1 (P < 0.00001); I^2^ = 98%). Test for subgroup differences for HIC versus LMIC: Chi^2^ = 53.13, df = 1 (P < 0.00001), I^2^ = 98.1%). We also carried out a sensitivity analysis to assess the impact of attrition on the outcome of satisfaction, showing no change in the estimate of the effect.

Contraceptive failure (unintended pregnancy) was reported in two studies (1129 women).

Risk Ratio (M–H, Fixed, 95% CI) was 0.47 [0.13, 1.67] (very low certainty evidence.

Only one woman reported serious adverse events in the three included trials (1261 women).

Risk Ratio (M–H, Fixed, 95% CI) was 0.34 [0.01, 8.22] (very low certainty evidence). These serious adverse events were menorrhagia and anemia requiring hospital admission) reported by one woman in the provider-administered group and resolved without sequelae.

Other adverse events were reported in two trials (863 women) with a Risk Ratio (M–H, Fixed, 95% CI) of 0.59 [0.28, 1.28] (very low certainty evidence). The other side effects included injection site pain or irritation, nausea, vomiting, irregular uterine bleeding, headaches, amenorrhea, decreased libido, and weight changes. All these non-serious adverse events did not require hospital treatment.

Details of GRADE summary of findings table all outcomes is shown in Table 2.

**Table 2:** Self-administration compared to provider-administered for DMPA-SC to improve contraceptive outcomes

Outcomes^$^	No. of participants (studies)	Certainty of the evidence (GRADE)	Relative effect (95% CI)	Anticipated absolute effects	
				Risk with provider-administered	Risk difference with self-administration
Continuation: all studies	1261 (3 RCTs)	⨁⨁⨁◯ MODERATE^a,b^	RR 1.35 (1.10–1.66)	460 per 1000	161 more per 1000 (46 more to 304 more)
Continuation—LMIC	731 (1 RCT)	⨁⨁◯◯ LOW^b,c^	RR 1.59 (1.40–1.81)	458 per 1000	270 more per 1000 (183 more to 371 more)
Continuation—HIC	530 (2 RCTs)	⨁◯◯◯ VERY LOW^a,b,d^	RR 1.22 (1.04–1.43)	463 per 1000	102 more per 1000 (19 more to 199 more)
Satisfaction—LMIC	731 (1 RCT)	⨁⨁◯◯ LOW^b,c,e^	RR 1.83 (1.61–2.07)	447 per 1000	371 more per 1000 (273 more to 478 more)
Satisfaction—HIC	398 (1 RCT)	⨁◯◯◯ VERY LOW^a,b,c,e^	RR 0.95 (0.84–1.07)	730 per 1000	37 fewer per 1000 (117 fewer to 51 more)
Pregnancy	1129 (2 RCTs)	⨁◯◯◯ VERY LOW^a,b,d,f^	RR 0.47 (0.13–1.67)	12 per 1000	7 fewer per 1000 (11 fewer to 8 more)
Serious adverse events	1261 (3 RCTs)	⨁◯◯◯ VERY LOW^a,b,d,f,g^	RR 0.34 (0.01–8.22)	2 per 1000	1 fewer per 1000 (2 fewer to 12 more)
Other adverse events	863 (2 RCTs)	⨁◯◯◯ VERY LOW^a,b,d,f,g^	RR 0.59 (0.28–1.28)	41 per 1000	17 fewer per 1000 (30 fewer to 12 more)

*Patient or population*: women using DMPA-SC for contraception; *Setting*: Outpatient; *Intervention*: Self-administration; *Comparison*: provider-administered

*The risk in the intervention group (and its 95% confidence interval) is based on the assumed risk in the comparison group and the *relative effect* of the intervention (and its 95% CI)

^$^Outcomes assessed with: Patient reported. Follow up: mean 12 months

*CI* confidence interval, *RR* risk ratio

GRADE Working Group grades of evidence: *High certainty* We are very confident that the true effect lies close to that of the estimate of the effect; *Moderate certainty* We are moderately confident in the effect estimate: The true effect is likely to be close to the estimate of the effect, but there is a possibility that it is substantially different; *Low certainty* Our confidence in the effect estimate is limited: The true effect may be substantially different from the estimate of the effect; *Very low certainty* We have very little confidence in the effect estimate: The true effect is likely to be substantially different from the estimate of effect

Explanations: ^a^Two studies had a potential source of selection bias related to the specific study design used: no blinding with a fixed block size

^b^Neither participants nor study staff were masked due to the nature of interventions. We judge that the performance is likely to be influenced by lack of blinding

^c^Data from one study and optimal information size not fulfilled

^d^The number of participants does not reach the optimal information size

^e^No blinding of outcome assessor. We judge that the outcome measurement is likely to be influenced by lack of blinding

^f^Wide confidence interval encompassing large effect size and no effect

^g^Data from studies with rare events

## Discussion

This review included three RCTs (1264 participants) that compared self-administered with provider administered DMPA-SC contraception. Self-administration, compared to provider-administration, significantly increased continuation. It was not possible to estimate an overall effect for women satisfaction, due to considerable heterogeneity with different direction of the effect estimate. There was no significant difference for unintended pregnancy, serious adverse events, and other adverse events (very low certainty evidence).

The main results suggest that self-administration, compared to provider-administration, probably improves continuation of DMPA-SC contraceptive method use. The effect size is larger in LMIC than in HIC and in younger than older women.

It is difficult to present a general effect estimate for women satisfaction because of multiple factors. First, only two trials reported satisfaction. Second, women reporting this outcome were not masked, obviously due to the nature of intervention. Considerable heterogeneity was detected with different effect. Test for subgroup differences, LMIC versus HIC, was significant. Consequently, we did not pool the data from these 2 studies.

We are uncertain about the effect on unintended pregnancy and adverse events because evidence was very low certainty. Only one woman in the three studies reported a serious adverse event, probably not related to the route of injection.

The three Included trials were at high risk of performance bias due to the lack of blinding of participants and personnel. The lack of blinding may also introduce detection bias at the outcome level for self-reported satisfaction. Further, the use of fixed size block randomization in two studies [15, 17] coupled with the lack of blinding may have introduced selection bias.

We considered heterogeneity induced by country level (HIC vs. LMIC). Test for subgroup differences was significant and explained substantial heterogeneity in continuity and satisfaction. We decided to combine the data and report totals and subtotals in subgroup meta‐analyses for continuity along with a sensitivity analysis of the model. We did not combine data for satisfaction.

We made an a‐priori decision to separate pooled estimates of the effect of self-administration on continuation for HIC and LMIC. Availability and accessibility differ and would result in larger and differential effects of self-administration in LMIC compared to HIC. Subgroup analyses of studies showed a larger effect of self‐administration in LMIC. Regarding satisfaction, we did not combine the 2 studies (one from HIC and the other from LMIC) because of considerable heterogeneity. Estimates differed in direction and providing an average from a random effect mode would have been misleading.

### Potential biases in the review process

We worked to reduce potential publication bias by conducting an extensive search without language restriction in major electronic databases and by scanning references of identified reviews and included studies. Nevertheless, we cannot rule out the possibility that we have missed relevant studies that were not published. Not all data needed to perform a full effect‐modifier investigation could be extracted from the published reports or revealed from the original authors. Thus, differences in age and education might modify or confound our reported estimates. We have not assessed the cost, ease of access, or the time to receive the contraception. This is important for program managers and policy makers.

### Agreements and disagreements with other studies or reviews

Other previously published reviews attempted to answer questions of safety, effectiveness of injectable contraceptive with different scopes [18–21].

We focused on DMPA-SC. We asked a question that is relevant to clinicians and women with participant-oriented outcomes. We restricted our eligibility criteria to RCTs. We regard this stringent criterion critical to mitigate the potential selection bias in included studies. Potential performance and detection bias were a matter of concern in all studies because blinding was not possible due to the nature of the interventions. Adding observational studies to the body of evidence would further lower our confidence in the estimates without a clear justification for including such studies. Further, in contrast to other reviews, clinical and methodological heterogeneity between studies have been carefully considered. This distinguishes our systematic review and its associated conclusions from previous ones.

### Implications for practice

The time-honored code of conduct in healthcare, “first do no harm” cannot be overstated when drafting implications for practice especially in areas like family planning.

With proper training in injection technique and schedule of administration, women may self-inject with DMPA-SC if their healthcare provider determines that it is appropriate, according to medical eligibility criteria [1], and with medical follow-up, as necessary [22].

Subcutaneous self-injection is practiced and is safe in other drugs such as insulin and heparin without serious risks attributed to self-administration [23].

Self-injection with DMPA SC enhances privacy and confidentiality. It gives women decision-making power over reproductive choices. Self-injection might be important in certain humanitarian conditions, where access to health care facility may be challenging, for example during adverse weather conditions, in displacement and when people are on the move. Expanding coverage of self-administered DMPA-SC may increase patient-centeredness and accessibility of contraception as well as reduce patient anxiety around COVID-19 transmission without losing contraceptive access [24].

Healthcare providers and program managers can add the option for DMPA-SC self-injection by women when considered appropriate by a healthcare professional. Self-injection of DMPA-SC may have a favorable effect on health economic and implementation outcomes [24–26].

## Conclusions

Self-administration of DMPA-SC, as compared to provider administration, probably improves continuation of contraceptive use. We are uncertain about the effects on satisfaction, contraceptive failure, and adverse events because of a very low certainty evidence. Further studies are still required regarding self-administered DMPA-SC.

## Supplementary Information

**Additional file 1.** Full detailed search strategy, and its translation to different databases, Full methods for the assessment of risk of bias, and Detailed risk of bias assessment for the included trials.

## Data Availability

Template data collection forms, data extracted from included studies, and data used for all analyses are available by contacting the corresponding author Professor Ashraf Nabhan.

## Abbreviations

CI: Confidence intervals
DMPA: Depot medroxyprogesterone acetate
DMPA-SC: Subcutaneous depot medroxyprogesterone acetate
GRADE: Grading of Recommendations, Assessment, Development and Evaluation
HIC: High income countries
LMIC: Low and Middle Income Countries
MPA: Medroxyprogesterone acetate
NNT: Number needed to treat
RCTs: Randomized controlled trials
RR: Risk ratio
