# Supplementary material for: Self-administered subcutaneous medroxyprogesterone acetate for improving contraceptive outcomes: a systematic review and meta-analysis
Source: BMC Womens Health. 2021 Oct 9;21:359. doi: 10.1186/s12905-021-01495-y (PMC8502084; doi:10.1186/s12905-021-01495-y)
Supplement: Supplementary file 1 — Additional file 1. Full detailed search strategy, and its translation to different databases, Full methods for the assessment of risk of bias, and Detailed risk of bias assessment for the included trials. [file 12905_2021_1495_MOESM1_ESM.docx]

Supplementary file 1

1. Full detailed search strategy, and its translation to different databases
2. Full methods for the assessment of risk of bias
3. Detailed risk of bias assessment for the included trials

Generic Search strategy

| line | Query |
| --- | --- |
| 1 | Medroxyprogesterone[Text Word] |
| 2 | Depo-Medroxyprogesterone[Text Word] |
| 3 | "Depo Medroxyprogesterone"[Text Word] |
| 4 | Depo-Provera[Text Word] |
| 5 | "Depo Provera"[Text Word] |
| 6 | Provera[Text Word] |
| 7 | Sayana[Text Word] OR "Depo subQ Provera"[Text Word] OR dmpa[Text Word] |
| 8 | Medroxyprogesterone[MeSH Terms] |
| 9 | Self Administration[MeSH Terms] |
| 10 | self[Text Word] |
| 11 | randomized controlled trial [pt] |
| 12 | controlled clinical trial [pt] |
| 13 | clinical trials as topic [mesh: noexp] |
| 14 | randomized [tiab] |
| 15 | placebo [tiab] |
| 16 | randomly [tiab] |
| 17 | trial [ti] |
| 18 | (animals [mh] NOT humans [mh]) |
| 19 | #1 \| #2 \| #3 \| #4 \| #5 \| #6 \| #7 \| #8 |
| 20 | #9 \| #10 |
| 21 | #11 \| #12 \| #13 \| #14 \| #15 \| #16 \| #17 |
| 22 | #21 NOT #18 |
| 23 | #19 AND #20 AND #22 |

# Translation to different databases

## CENTRAL

(Medroxyprogesterone:ti,ab,kw OR Depo-Medroxyprogesterone:ti,ab,kw OR "Depo Medroxyprogesterone":ti,ab,kw OR Depo-Provera:ti,ab,kw OR "Depo Provera":ti,ab,kw OR Provera:ti,ab,kw OR Sayana:ti,ab,kw OR "Depo subQ Provera":ti,ab,kw OR dmpa:ti,ab,kw OR [mh Medroxyprogesterone])

AND

([mh "Self Administration"] OR self:ti,ab,kw)

## CINAHL

(Medroxyprogesterone OR Depo-Medroxyprogesterone OR "Depo Medroxyprogesterone" OR Depo-Provera OR "Depo Provera" OR Provera OR Sayana OR "Depo subQ Provera" OR dmpa OR (MH "Medroxyprogesterone+"))

AND

((MH "Self Administration+") OR self)

AND

((PT "randomized controlled trial" OR PT "controlled clinical trial" OR (MH "clinical trials as topic") OR TI randomized OR AB randomized OR TI placebo OR AB placebo OR TI randomly OR AB randomly OR TI trial) NOT ((MH "animals +") NOT (MH "humans +")))

## Embase

(Medroxyprogesterone:ti,ab,de,tn OR Depo-Medroxyprogesterone:ti,ab,de,tn OR "Depo Medroxyprogesterone":ti,ab,de,tn OR Depo-Provera:ti,ab,de,tn OR "Depo Provera":ti,ab,de,tn OR Provera:ti,ab,de,tn OR Sayana:ti,ab,de,tn OR "Depo subQ Provera":ti,ab,de,tn OR dmpa:ti,ab,de,tn OR 'Medroxyprogesterone'/exp)
AND
('Self Administration'/exp OR self:ti,ab,de,tn)
AND
(("randomized controlled trial":it OR "controlled clinical trial":it OR 'clinical trials as topic '/de OR randomized:ti,ab OR placebo:ti,ab OR randomly:ti,ab OR trial:ti) NOT ('animals '/exp NOT 'humans '/exp))

## MEDLINE (Ovid)

(Medroxyprogesterone.mp. OR Depo-Medroxyprogesterone.mp. OR Depo Medroxyprogesterone.mp. OR Depo-Provera.mp. OR Depo Provera.mp. OR Provera.mp. OR Sayana.mp. OR Depo subQ Provera.mp. OR dmpa.mp. OR exp Medroxyprogesterone/)

AND

(exp Self Administration/ OR self.mp.)

AND

((randomized controlled trial .pt. OR controlled clinical trial .pt. OR clinical trials as topic / OR randomized .ti,ab. OR placebo .ti,ab. OR randomly .ti,ab. OR trial .ti.) NOT (exp animals / NOT exp humans /))

## Web of Science

(Medroxyprogesterone OR Depo-Medroxyprogesterone OR "Depo Medroxyprogesterone" OR Depo-Provera OR "Depo Provera" OR Provera OR Sayana OR "Depo subQ Provera" OR dmpa OR Medroxyprogesterone)

AND

("Self Administration" OR self)

AND

(("randomized controlled trial" OR "controlled clinical trial" OR "clinical trials as topic" OR randomized OR placebo OR randomly OR trial) NOT (animals NOT humans))

## Scopus

(TITLE-ABS-KEY("Medroxyprogesterone") OR TITLE-ABS-KEY("Depo-Medroxyprogesterone") OR TITLE-ABS-KEY("Depo Medroxyprogesterone") OR TITLE-ABS-KEY("Depo-Provera") OR TITLE-ABS-KEY("Depo Provera") OR TITLE-ABS-KEY("Provera") OR TITLE-ABS-KEY("Sayana") OR TITLE-ABS-KEY("Depo subQ Provera") OR TITLE-ABS-KEY("dmpa") OR INDEXTERMS("Medroxyprogesterone"))

AND

(INDEXTERMS("Self Administration") OR TITLE-ABS-KEY("self"))

AND

((DOCTYPE("randomized controlled trial ") OR DOCTYPE("controlled clinical trial ") OR INDEXTERMS("clinical trials as topic ") OR TITLE-ABS("randomized ") OR TITLE-ABS("placebo ") OR TITLE-ABS("randomly ") OR TITLE("trial ")) NOT (INDEXTERMS("animals ") NOT INDEXTERMS("humans ")))

# Pre-specified Methods for Risk of bias assessment

1. RANDOM SEQUENCE GENERATION (CHECKING FOR POSSIBLE SELECTION BIAS): We will describe for each included study the method used to generate the allocation sequence in sufficient detail to allow an assessment of whether it should produce comparable groups. We will assess the method as:
   1. Low risk (any truly random process, e.g. random number table; computer random number generator);
   2. High risk (any non-random process, e.g. odd or even date of birth; hospital or clinic record number); or
   3. Unclear risk.
2. ALLOCATION CONCEALMENT (CHECKING FOR POSSIBLE SELECTION BIAS): We will describe for each included study the method used to conceal the allocation sequence and determine whether intervention allocation could have been foreseen in advance of, or during recruitment, or changed after assignment. We will assess the method as:
   1. Low risk (e.g. telephone or central randomisation; consecutively-numbered sealed opaque envelopes);
   2. High risk (open random allocation; unsealed or non-opaque envelopes); or
   3. Unclear risk.
3. Blinding
   1. BLINDING OF PARTICIPANTS AND PERSONNEL (CHECKING FOR POSSIBLE PERFORMANCE BIAS): The nature of the intervention does not allow for blinding of participants. We will consider that studies are at low risk of bias if we judge that the lack of blinding could not have affected the results.
   2. BLINDING OF OUTCOME ASSESSMENT (CHECKING FOR POSSIBLE DETECTION BIAS): We will describe all measures used, if any, to blind outcome assessors from knowledge of which intervention a participant received and provide any information relating to whether the intended blinding was effective.
   3. We will assess the method as Low risk; High risk; or Unclear risk.
4. INCOMPLETE OUTCOME DATA (CHECKING FOR POSSIBLE ATTRITION BIAS): We will describe for each included study, and for each outcome or class of outcomes, the completeness of data including attrition and exclusions from the analysis. We will state whether attrition and exclusions were reported, the numbers included in the analysis at each stage (compared with the total randomised participants), reasons for attrition or exclusion where reported, and whether missing data were balanced across groups or were related to outcomes. Where sufficient information is reported, or can be supplied by the trial authors, we will re-include missing data in the analyses which we undertake. We will assess methods as:
   1. Low risk;
   2. High risk; or
   3. Unclear risk.
5. SELECTIVE REPORTING BIAS: We will describe for each included study how we investigated the possibility of selective outcome reporting bias and what we found. We will assess the methods as:
   1. Low risk (where it is clear that all of the study’s prespecified outcomes and all expected outcomes of interest to the review have been reported);
   2. High risk (where not all the study’s prespecified outcomes have been reported; one or more reported primary outcomes were not prespecified; outcomes of interest are reported incompletely and so cannot be used; the study fails to include results of a key outcome that would have been expected to have been reported); or
   3. Unclear risk.
6. OTHER SOURCES OF BIAS: We will describe for each included study any important concerns we have about other possible sources of bias. We will assess whether each study was free of other problems that could put it at risk of bias:
   1. Low risk;
   2. High risk; or
   3. Unclear risk.
7. Overall risk of bias: We will make explicit judgements about the overall risk of bias i.e. whether studies are at high risk of bias, according to the criteria given in the Cochrane Handbook.

Detailed risk of bias assessment and justification for included studies

Beasley 2014

Risk of bias table

| Bias | Authors' judgement | Support for judgement |
| --- | --- | --- |
| Random sequence generation (selection bias) | Low risk | "a computerized random-number generator in blocks of six" "An investigator not involved with participant contact generated the allocation schedule" |
| Allocation concealment (selection bias) | Low risk | "sequentially numbered opaque envelopes" |
| Blinding of participants and personnel (performance bias) | High risk | "Neither participants nor study staff were masked" due to the nature of interventions. We judge that the performance is likely to be influenced by lack of blinding |
| Blinding of outcome assessment (detection bias) Continuation, pregnancy, adverse events | Low risk | No blinding of outcome assessor. We judge that the outcome measurement is not likely to be influenced by lack of blinding. |
| Blinding of outcome assessment (detection bias) Satisfaction | High risk | No blinding of outcome assessor. We judge that the outcome measurement is likely to be influenced by lack of blinding |
| Incomplete outcome data (attrition bias) | Low risk | One hundred fifteen women (87%) completed 12 months follow-up. Ten participants (11.6%) in the self-administration group and six (13.0%) in the clinic administration were lost to follow up. Trialists assumed that all participants who were lost to follow-up had discontinued DMPA use. One participant (2.1%) in the clinic administration group withdrew due to a desire to get pregnant. |
| Selective reporting (reporting bias) | Low risk | All of the study’s pre-specified (primary and secondary) outcomes that are of interest in the review have been reported in the pre-specified way. |
| Other bias | High risk | Had a potential source of bias related to the specific study design used: no blinding with a fixed block size. |

Burke 2018

Risk of bias table

| Bias | Authors' judgement | Support for judgement |
| --- | --- | --- |
| Random sequence generation (selection bias) | Low risk | a computer-generated block randomisation schedule with block sizes of four, six, and eight and stratification by study site. |
| Allocation concealment (selection bias) | Low risk | sequentially numbered opaque envelopes. The randomisation schedule and envelopes were prepared by an independent statistician who was not otherwise involved in the study. |
| Blinding of participants and personnel (performance bias) | High risk | "Neither participants nor study staff were masked" due to the nature of interventions. We judge that the performance is likely to be influenced by lack of blinding |
| Blinding of outcome assessment (detection bias) Continuation, pregnancy, adverse events | Low risk | No blinding of outcome assessor. We judge that the outcome measurement is not likely to be influenced by lack of blinding |
| Blinding of outcome assessment (detection bias) Satisfaction | High risk | No blinding of outcome assessor. We judge that the outcome measurement is likely to be influenced by lack of blinding |
| Incomplete outcome data (attrition bias) | Low risk | intention-to-treat analysis |
| Selective reporting (reporting bias) | Low risk | All of the study’s pre-specified (primary and secondary) outcomes that are of interest in the review have been reported in the pre-specified way; |
| Other bias | Low risk | The study appears to be free of other sources of bias. |

Kohn 2018

Risk of bias table

| Bias | Authors' judgement | Support for judgement |
| --- | --- | --- |
| Random sequence generation (selection bias) | Low risk | a random number generator in blocks of six |
| Allocation concealment (selection bias) | Low risk | individual assignments were placed in sequentially numbered opaque envelopes |
| Blinding of participants and personnel (performance bias) | High risk | "Neither participants nor study staff were masked" due to the nature of interventions. We judge that the performance is likely to be influenced by lack of blinding |
| Blinding of outcome assessment (detection bias) Continuation, pregnancy, adverse events | Low risk | No blinding of outcome assessor. We judge that the outcome measurement is not likely to be influenced by lack of blinding |
| Blinding of outcome assessment (detection bias) Satisfaction | High risk | No blinding of outcome assessor. We judge that the outcome measurement is likely to be influenced by lack of blinding |
| Incomplete outcome data (attrition bias) | Low risk | Allocated to CLINIC administration (n=200), Allocated to SELF administration (n=201). 336 participants (84%) completed the 12-month survey. Response rates and loss-to-follow-up were similar between study arms |
| Selective reporting (reporting bias) | Low risk | All of the study’s pre-specified (primary and secondary) outcomes that are of interest in the review have been reported in the pre-specified way; |
| Other bias | High risk | Had a potential source of bias related to the specific study design used: no blinding with a fixed block size |
